# Femoral Fixation Techniques for Lateral Extra-Articular Tenodesis During Anterior Cruciate Ligament Reconstruction: A Systematic Review and Proposed Collision-Aware Planning Framework

**DOI:** 10.3390/medicina62081542

**Published:** 2026-08-11

**Authors:** Nifon K. Gkekas, Antonis Sergiou, Angelo V. Vasiliadis, Vasileios Akrivos, Evangelos Gatos, Michael Hantes

**Affiliations:** 1 Department of Orthopaedic Surgery & Musculoskeletal Trauma, Faculty of Medicine, School of Health Sciences, University of Thessaly, 41500 Larissa, Greece; nifwn_gekas@hotmail.com (N.K.G.); vasilisakrivos21@gmail.com (V.A.); vaggatos@gmail.com (E.G.); 2 Department of Orthopaedic Surgery, Sports Trauma Unit, St. Luke’s Hospital, K. Varnali 44, 55534 Thessaloniki, Greece; vasiliadis.av@gmail.com

**Keywords:** anterior cruciate ligament reconstruction, lateral extra-articular tenodesis, femoral fixation, tunnel convergence, tunnel collision, biomechanics

## Abstract

*Background and Objectives:* Lateral extra-articular tenodesis (LET) is increasingly used to augment anterior cruciate ligament reconstruction (ACLR) in selected high-risk patients. Femoral fixation strategies vary widely and may influence construct behavior, tunnel interaction, technical safety, and ultimately knee stability and functional outcomes. The objectives of this review were to map and compare femoral fixation techniques for LET performed with ACLR, to summarize the cadaveric and clinical evidence relevant to fixation choice, to synthesize imaging and experimental data on femoral tunnel convergence and collision-prevention strategies, and to propose an evidence-informed, collision-aware planning framework for femoral fixation selection. *Materials and Methods:* A systematic review with narrative synthesis was conducted in accordance with the Preferred Reporting Items for Systematic Reviews and Meta-Analyses (PRISMA) 2020 guidelines. PubMed/MEDLINE, Embase, and the Cochrane Library were searched from inception through February 2026. Eligible studies were classified a priori as direct LET clinical evidence, LET biomechanical evidence, LET imaging or convergence evidence, extrapolated anterolateral ligament (ALL) or broader lateral augmentation tunnel-geometry evidence, technical guidance, or contextual background. *Results:* Thirty-three studies met the eligibility criteria and provided extractable fixation-specific, biomechanical, imaging, or technical data. Femoral fixation techniques clustered into inlay tunnel or socket fixation, onlay cortical fixation, suspensory or indirect concepts, and alternative designs. Biomechanical and imaging studies demonstrated construct-specific differences in restraint behavior, overconstraint signals, tunnel trajectory, and convergence risk. Clinical studies reported fixation-related variation in complications and failures, although comparative fixation-specific clinical evidence remained limited and heterogeneous. *Conclusions:* Available biomechanical and imaging evidence suggests that the femoral fixation family, implant geometry, tunnel trajectory, and graft fixation angle may influence construct behavior and tunnel safety; however, direct comparative clinical evidence remains limited, and these observations should be interpreted with caution. The fixation taxonomy and collision-aware planning framework proposed here are intended as a structured, evidence-informed aid for describing and planning femoral fixation strategies rather than as a validated decision tool, and they highlight the need for standardized reporting of the fixation family, entry point, drilling trajectory, tunnel or socket dimensions, fixation angle, and collision-control strategy.

## 1. Introduction

Lateral extra-articular tenodesis (LET) is an extra-articular lateral augmentation procedure performed together with anterior cruciate ligament reconstruction (ACLR) to improve control of anterolateral rotatory laxity, reduce pivot shift, and potentially decrease graft overload in selected high-risk patients. Common techniques include modified Lemaire, MacIntosh, Coker-Arnold, and Ellison-type procedures; these differ in graft passage, fixation location, and surgical workflow [1,2]. Importantly, the named LET procedure and the femoral fixation method are related but not identical variables: the same general LET concept may be performed using different femoral fixation families, implants, and fixation angles [3].

LET is most often considered in young or athletic patients, revision settings, high-grade pivot shift, generalized laxity, high-risk pivoting sports, or other settings in which graft failure risk is clinically important [1]. The STABILITY randomized clinical trial reported that adding LET to hamstring autograft ACLR reduced graft failure in young active patients, and contemporary reviews of lateral extra-articular procedures and of graft-failure prevention similarly identify lateral augmentation as a strategy to consider in high-risk athletes [2,3,4]. Biomechanical evidence supports LET as a secondary restraint of tibial internal rotation, with cadaveric and robotic studies confirming improved rotational stability and a load-sharing effect on the ACL graft [5,6,7]. Long-term radiographic data further support its protective role: a recent meta-analysis demonstrated that combined ACLR and LET significantly reduces moderate-to-severe lateral compartment osteoarthritis compared with isolated ACLR, with the effect most pronounced following meniscectomy [8].

Femoral fixation is the most variable technical component of LET. Fixation choice determines tunnel burden, implant profile, and tensioning control, and available evidence suggests that constructs are not interchangeable and that the fixation protocol, particularly knee flexion angle at the time of graft fixation, materially influences restraint behavior [9,10,11,12,13]. Femoral tunnel convergence is a clinically relevant risk when lateral augmentation is combined with ACLR, determined by modifiable factors including entry point, trajectory, and drilling angle, and documented in both cadaveric and in vivo imaging studies [14,15,16,17].

The primary objectives of this systematic review with narrative synthesis were to map and compare femoral fixation techniques used for LET performed with ACLR and to propose a collision-aware planning framework for femoral fixation family selection. Secondary objectives were to summarize the cadaveric and clinical evidence relevant to fixation choice and to synthesize the available evidence on femoral tunnel convergence, tunnel collision, and prevention strategies.

## 2. Materials and Methods

### 2.1. Protocol and Registration

This systematic review was registered in the International Prospective Register of Systematic Reviews (PROSPERO; registration number CRD420261396698) and was conducted in accordance with the Preferred Reporting Items for Systematic Reviews and Meta-Analyses (PRISMA) 2020 guidelines [18]. The completed PRISMA 2020 checklist is provided in Supplementary Table S1.

### 2.2. Search Strategy

A systematic search was performed in PubMed/MEDLINE, Embase, and the Cochrane Library from inception through February 2026. No lower date limit was applied, so that both foundational and the most recent studies would be captured. The full electronic search strategy for all databases, including the exact Boolean syntax, search date, database platform, filters, and database-specific adaptations, is provided in Supplementary Table S2. The PubMed core search string was: ((“lateral extra-articular tenodesis” OR LET OR “modified Lemaire” OR Lemaire OR MacIntosh OR “Coker-Arnold” OR “anterolateral ligament” OR “anterolateral augmentation” OR ALL) AND (“anterior cruciate ligament reconstruction” OR “ACL reconstruction” OR ACLR) AND (“femoral fixation” OR fixation OR tunnel OR socket OR “tunnel convergence” OR “tunnel collision” OR biomechanical OR cadaveric OR CT OR “computed tomography”)). Reference lists of all included studies were hand-searched for additional eligible articles.

### 2.3. Eligibility Criteria

Studies were eligible if they evaluated ACLR combined with LET or related anterolateral augmentation and provided extractable data on at least one of the following domains: femoral fixation construct or protocol, biomechanical outcomes, clinical outcomes or fixation-related complications, or femoral tunnel convergence and mitigation strategies. Eligible study designs included clinical studies, cadaveric or biomechanical studies, computed tomography (CT) or three-dimensional imaging studies, and technical reports with direct relevance to femoral fixation or tunnel safety. Case reports, editorials, and commentaries were excluded. Only English-language publications were included.

### 2.4. Evidence Classification and Data Extraction

Evidence categories were defined a priori. Direct LET clinical and biomechanical studies were used for LET-specific inference. Anterolateral ligament (ALL) reconstruction or broader anterolateral augmentation studies were used only for tunnel-geometry extrapolation when the femoral tunnel corridor, drilling direction, or convergence mechanism was directly relevant to combined ACLR and lateral augmentation. Technical reports were used for operative feasibility, landmark description, and collision-avoidance strategies, but not for comparative clinical efficacy. Extracted data were tabulated in a structured evidence table (Table 1, Table S3).

### 2.5. Risk of Bias and Quality Assessment

Risk of bias and methodological quality were assessed for all included studies. The methodological quality of every included empirical study—clinical, cadaveric, biomechanical, and imaging—was appraised using the Methodological Index for Non-Randomized Studies (MINORS), a validated instrument for non-randomized surgical and experimental studies that is applicable to both clinical and cadaveric or laboratory interventions. Non-comparative studies were scored against the eight core MINORS items (ideal score 16) and comparative studies against all twelve items (ideal score 24). For clinical studies, the level of evidence was additionally categorized according to the Oxford Centre for Evidence-Based Medicine (OCEBM) Levels of Evidence. Purely descriptive technical notes, which report an operative technique rather than a study outcome, are not amenable to MINORS scoring and were instead appraised for reproducibility of landmarks, implant description, tunnel or implant trajectory, and explicit collision-avoidance strategy. The complete methodological quality appraisal is presented in Table 2. This appraisal was used to interpret the strength and certainty of the synthesized statements rather than to exclude studies.

### 2.6. Data Synthesis

Formal meta-analysis was considered for graft failure, LET-related complications, hardware irritation or removal, tunnel convergence, and load-to-failure outcomes, but pooling was not feasible because outcomes, denominators, fixation families, and measurement methods were inconsistently reported. Therefore, results were synthesized narratively and organized by fixation family and evidence role.

## 3. Results

### 3.1. Study Selection

**Figure 1:**
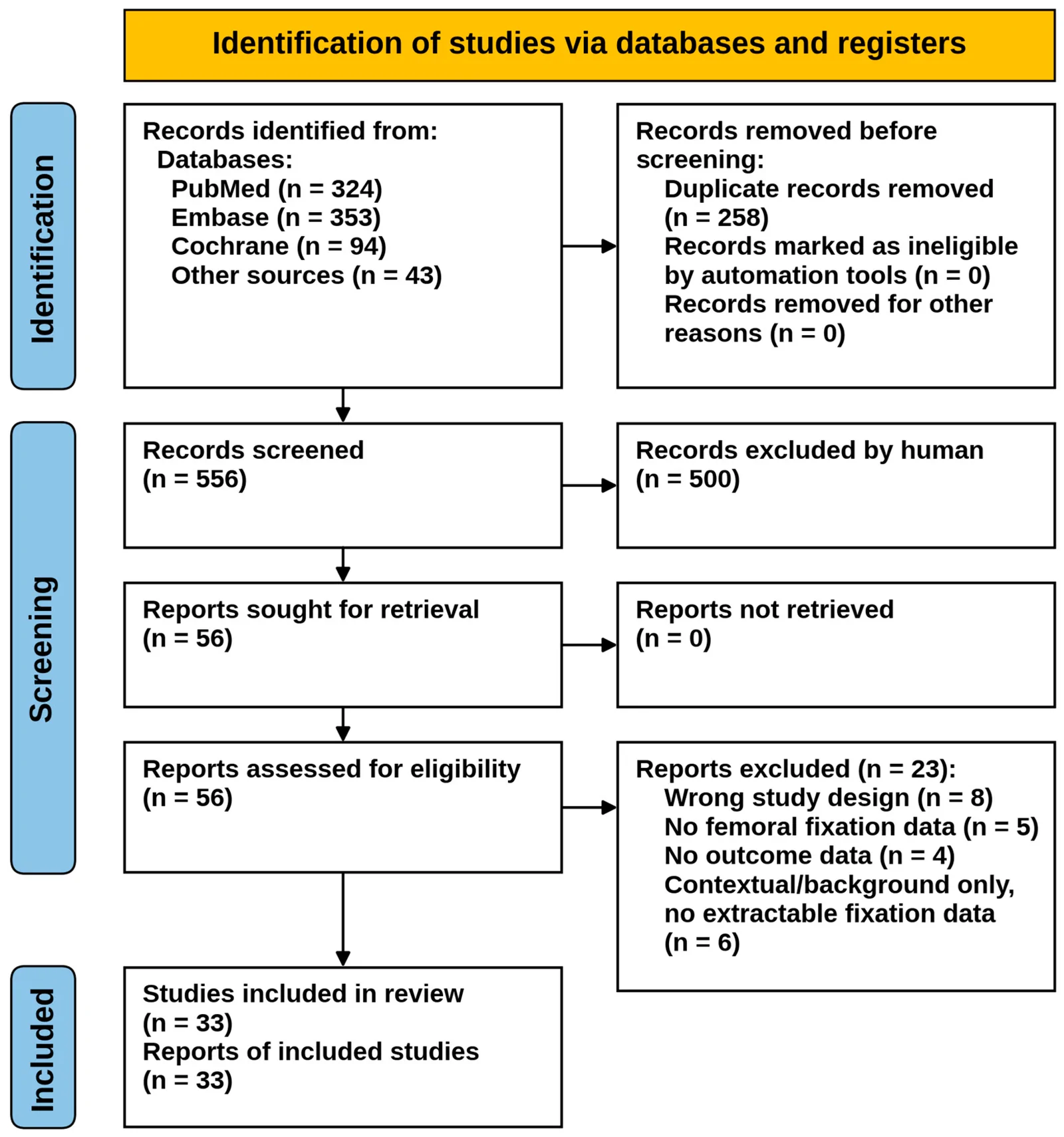
PRISMA 2020 flow diagram for the systematic review of femoral fixation techniques for lateral extra-articular tenodesis during anterior cruciate ligament reconstruction. Records were identified from databases (PubMed, Embase, and the Cochrane Library) and other sources; record counts are shown at the identification, screening, and inclusion stages.

A total of 814 records were identified before deduplication: 771 through database searching (PubMed *n* = 324, Embase *n* = 353, Cochrane Library *n* = 94) and 43 through reference-list searching and other sources. The 43 additional records were imported into the same screening database before deduplication; therefore, the 556 records screened represent the combined deduplicated yield from databases and other sources. Screening was conducted independently by two reviewers, with disagreements resolved by consensus. Five hundred records were excluded at title and abstract screening. The remaining 56 reports advanced to full-text assessment. During full-text review, 23 reports were excluded: 8 for a study design not specifically addressing femoral fixation technique, 5 for insufficient data on femoral fixation construct or protocol details, 4 for absence of relevant clinical, biomechanical, imaging, or safety outcome data, and 6 for providing only contextual or background information without extractable fixation-specific data. A total of 33 studies met the final inclusion criteria and are summarized in Table 1. The study selection process is summarized in the PRISMA 2020 flow diagram (Figure 1).

The methodological quality of the included studies, appraised with the Methodological Index for Non-Randomized Studies (MINORS), is summarized in Table 2; comparative studies were scored against twelve items (ideal score 24) and non-comparative studies against eight items (ideal score 16), while purely descriptive technical notes were appraised narratively rather than scored.

### 3.2. Femoral Fixation Taxonomy

**Figure 2:**
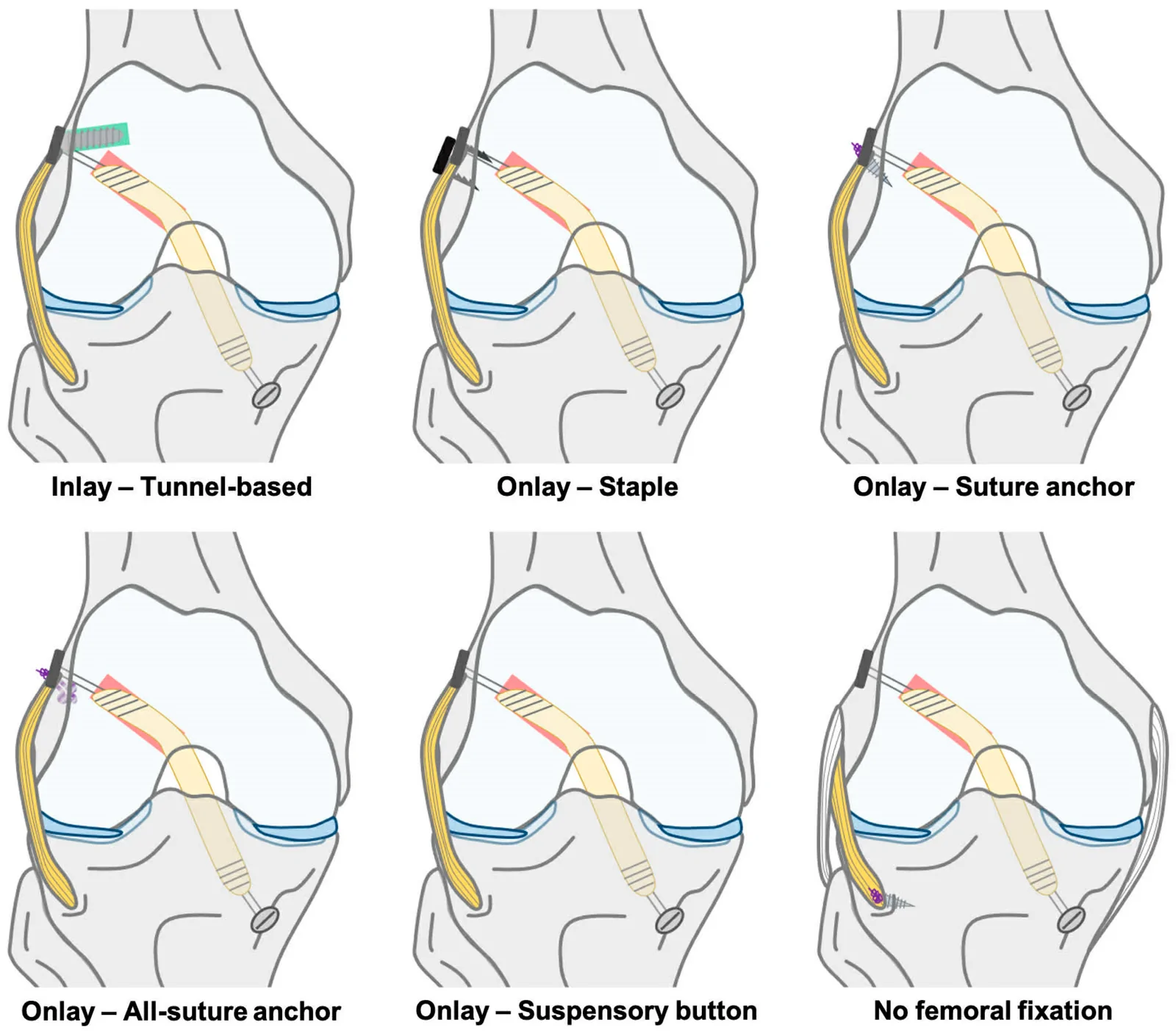
Schematic overview of the main femoral fixation families used in lateral extra-articular tenodesis (LET), including inlay tunnel or socket fixation, onlay cortical fixation, suspensory or indirect fixation concepts, and alternative designs.

Femoral fixation determines (a) whether a dedicated LET femoral tunnel or socket is created, (b) how tension is applied before final fixation, and (c) the likely interaction with the ACL femoral tunnel. The included literature supported a practical taxonomy: inlay fixation (tunnel or socket-based), onlay fixation (cortical surface-based), suspensory or indirect fixation concepts, and alternative designs (Figure 2). This framework links technique choice with the biomechanical evidence, clinical reporting, and tunnel convergence risk described in the sections that follow.

#### 3.2.1. Inlay Fixation: Tunnel or Socket-Based

Inlay fixation places the iliotibial band (ITB) strip within a femoral socket and secures it using an interference screw. This creates a compact construct and uses a familiar surgical workflow, but its biomechanical advantage over other fixation families should not be assumed. In the included cadaveric studies, inlay interference screw fixation was reported as biomechanically adequate and, in some comparisons, demonstrated higher load-to-failure than staple or anchor constructs [9,10]. Its key limitation is intrinsic: it requires an additional femoral tunnel in the same corridor as the ACL femoral tunnel. Tunnel planning and drilling trajectory therefore become central determinants of technical safety, particularly in anatomic ACLR where the ACL tunnel already occupies a large portion of the lateral femoral condyle. Intraoperative strategies to mitigate collision risk, including arthroscopic visualization of the ACL femoral tunnel during LET socket drilling, have been described by Mitrousias et al. [35].

#### 3.2.2. Onlay Fixation: Cortical Surface-Based

Onlay fixation secures the graft on the lateral femoral cortex without a dedicated LET tunnel, shifting technical priorities toward implant profile, cortical purchase, implant trajectory, and tensioning reproducibility. Onlay fixation reduces the need for a dedicated LET socket but does not eliminate tunnel hazards, because staples, anchors, or drilling trajectories can still threaten the ACL femoral tunnel [24].

Onlay fixation should be interpreted by implant subtype rather than as a single category. Xu et al. reported that cortical surface fixation produced less overconstraint than intra-tunnel constructs in deep flexion [11]. Staple-based onlay fixation is simple and widely used but carries a documented risk of ACL tunnel violation and hardware irritation or removal [24,44]. Anchor-based onlay fixation allows flexible placement and pre-fixation tension adjustment; Behrendt et al. reported comparable short-term outcomes between onlay anchor and transosseous fixation in revision ACLR with LET [21]. All-suture anchors offer a low-profile evolution with supportive biomechanical and small clinical-series evidence [9,22,23,26]. Technical reports have further described knotless anchor, all-suture anchor, and comparative onlay techniques for modified Lemaire LET [36,42,43].

#### 3.2.3. Suspensory and Indirect Fixation Concepts

Cortical suspensory button fixation combined with suture tape augmentation has been described for LET, enabling incremental tensioning [37]. Indirect concepts leverage the ACL femoral suspensory device to avoid a separate LET femoral implant: the ITB strip is fixed using ACL button sutures [38], or a common suspensory device is shared for both ACLR and modified Lemaire tenodesis [39]. These approaches are attractive for collision-conscious planning but introduce construct coupling considerations and currently have limited comparative validation.

#### 3.2.4. Alternative Designs

Distally fixed augmentation based on a modified Ellison concept avoids proximal femoral fixation and tunnel interaction by design. In robotic cadaveric testing, it reduced anterolateral rotational laxity and restored kinematics close to intact, with overconstraint signals limited to specific early flexion conditions [25]. Although not interchangeable with modified Lemaire LET in most workflows, it illustrates the principle of collision avoidance by design.

### 3.3. Cadaveric and Biomechanical Findings

Cadaveric and robotic biomechanical studies provided the most controlled evidence on how LET alters knee mechanics when combined with ACLR and how femoral fixation choices influence construct behavior. The available studies converged on three themes: (1) the stabilizing effect of LET on rotational control, (2) the structural properties and failure behavior of common fixation constructs, and (3) safety signals, particularly overconstraint and tunnel interaction.

#### 3.3.1. Biomechanical Contribution of Let Combined with Acl Reconstruction

Delaloye et al. and Geeslin et al. reported that adding LET or related lateral augmentation to ACLR reduces internal rotation laxity and improves control of pivot shift-related kinematics compared with ACLR alone [5,6]. Mayr et al. reported that modified Lemaire LET reduces ACL graft forces during internal tibial torque loading, supporting a load-sharing mechanism [7]. Restraint profiles, however, depended on the fixation protocol: Inderhaug et al. demonstrated that the knee flexion angle at fixation materially affects the restraint behavior of anterolateral procedures combined with ACLR and should therefore be treated as part of the fixation strategy rather than an operative detail [12].

#### 3.3.2. Biomechanical Performance by Femoral Fixation Method

Gilat et al. compared modified Lemaire LET fixation constructs and reported that interference screw and suture anchor fixation showed comparable load-to-failure profiles, whereas staple fixation performed less favorably [9]. Tollefson et al. reported adequate failure loads for interference screw, staple, and suture anchor constructs, suggesting that multiple fixation families may meet baseline strength requirements, although with differing failure characteristics [10]. Tejada et al. reported sufficient biomechanical stability for an all-suture anchor onlay construct [26]. Xu et al. reported that cortical surface fixation produced less overconstraint than intra-tunnel constructs in deep flexion [11]. Devitt et al. found that a distally fixed augmentation reduced anterolateral rotational laxity and restored kinematics close to intact, with overconstraint confined to specific early flexion conditions [25].

#### 3.3.3. Biomechanical Concerns and Safety Signals

Overconstraint could occur depending on the fixation location and protocol, particularly at higher flexion angles, supporting standardized reporting of the fixation angle [11,12]. Tunnel interaction remained a relevant safety concern. Zhu et al. provided cadaveric guidance on safe fixation depth and drilling orientation [27]. Jaecker et al. described radiographic landmarks to improve reproducibility of femoral tunnel positioning [34]. Kawanishi et al. confirmed that drilling angle materially affects collision risk in combined ACL and anterolateral tunnel procedures [30]. Critically, onlay fixation did not eliminate tunnel hazards; Moran et al. showed that staple fixation may penetrate the ACL reconstruction tunnel, emphasizing that implant placement remains critical even without a dedicated LET socket [24]. Single-tunnel concepts aiming to enhance rotational stability while limiting additional femoral tunnels have also been described by Manze et al. [40].

### 3.4. Clinical Findings

Clinical evidence supported LET as an adjunct to ACLR in selected high-risk patients, but fixation-specific reporting remained heterogeneous. Most studies evaluated LET as a concept rather than isolating the femoral fixation method.

#### 3.4.1. Fixation Method and Graft Failure

Johnson et al. synthesized the available clinical literature and reported that ACLR combined with LET is associated with lower primary graft failure than ACLR alone, while suggesting variation in failure rates across fixation methods including staple, suture anchor, and cortical or suspensory constructs [19]. Although heterogeneity in technique detail and reporting limited inference, this synthesis framed femoral fixation as a clinically relevant variable. Zabrzynski et al. documented substantial variability in fixation methods across published MacIntosh and modified Lemaire-based series, with interference screw, staple, and suture anchor fixation all commonly reported, limiting direct comparisons [20]. Kolin et al. further confirmed that the fixation angle is inconsistently documented across LET and related constructs and should be considered when interpreting clinical outcomes [13].

#### 3.4.2. Comparative Clinical Evidence Reporting Femoral Fixation Constructs

Direct clinical comparisons between fixation strategies remained limited. In revision ACLR, Behrendt et al. reported comparable short-term outcomes between onlay anchor fixation and transosseous fixation for LET, supporting multiple tunnel-sparing options when femoral tunnel conflict is a key constraint [21]. Conteduca et al. described a retrospective, non-comparative series of 15 patients undergoing revision ACLR and modified Lemaire tenodesis with a 2.6 mm knotless suture anchor, reporting favorable clinical and radiographic outcomes at a minimum of 2-year follow-up without major complications [22]. Erdmann, Zabrzynski et al. reported a small prospective series of 32 patients undergoing primary or revision ACLR with modified Lemaire LET using a self-punching all-suture anchor and mini-open technique, with improvements in Lysholm and IKDC scores at follow-up [23]. These studies support the feasibility of low-profile anchor and all-suture anchor fixation in small non-comparative or limited comparative series, but they do not establish clinical superiority over other fixation families. Complication profiles may differ across fixation families. Zabrzynski et al. identified nine distinct complication types after LET combined with ACLR across seven studies (*n* = 1121 patients), with reported rates ranging from 0.6% to 17%; the overall LET complication rate was 4.2%, corresponding to approximately 47 complications if the full denominator of 1121 patients is applied. Hardware irritation was the most frequent adverse event and was predominantly associated with staple fixation. Hardware removal should be interpreted as a secondary procedure or reoperation burden, and the denominator used for hardware removal should be reported separately when available [44].

### 3.5. Femoral Tunnel Convergence and Collision

Femoral tunnel convergence is a clinically relevant technical problem when lateral augmentation is combined with ACLR. In this review, tunnel convergence referred to the radiographic, intraoperative, or simulated approximation or intersection risk between the ACL femoral tunnel and a LET or anterolateral femoral tunnel or implant trajectory. Tunnel collision referred to actual communication, violation, or breach between tunnels or hardware paths. Because definitions varied across the included studies, convergence and collision outcomes were synthesized narratively rather than pooled. Risk is highest when a separate femoral tunnel is created within the limited lateral femoral corridor already occupied by the ACL femoral tunnel. Convergence or collision may compromise the bone bridge, weaken fixation, complicate revision planning, or directly violate the ACL tunnel (Table S4).

#### 3.5.1. Mechanisms and Determinants

Convergence is primarily determined by three modifiable factors: (a) the relative femoral tunnel entry points, (b) tunnel trajectory in the axial and coronal planes, and (c) knee flexion angle during tunnel creation. Jaecker et al. confirmed high convergence risk in combined ACLR and LET, reflecting the narrow femoral corridor and the sensitivity of collision risk to small changes in placement [14]. Smeets et al. reported similar tunnel-geometry concerns for combined ACL and ALL reconstruction [15]. ALL tunnel studies were included only when their findings informed lateral femoral tunnel geometry; they were not used to infer clinical superiority of LET fixation methods.

#### 3.5.2. Incidence in Clinical and Imaging Studies

CT-based studies confirmed that tunnel interaction occurs in vivo. Perelli et al. specifically evaluated ACLR with modified Lemaire tenodesis and reported that drilling angle is a modifiable determinant of LET tunnel convergence risk [16]. Suh et al. reported tunnel convergence patterns in 227 patients undergoing combined ACL and ALL reconstruction [17]. These ALL data are relevant to the limited lateral femoral corridor and to drilling-direction principles, but they are not anatomically identical to modified Lemaire LET because graft path, entry point, fixation depth, and implant type differ. Clinical positioning data reinforced this picture. Kanakamedala et al. reported high variability in LET femoral tunnel position with landmark-based techniques, with a substantial proportion of fixation points outside a predefined radiographic target zone, contributing to variability in restraint profiles and tunnel interaction risk [31]. Kittl et al. confirmed that convergence rates in combined ACL and anterolateral structure reconstructions are influenced by ACL knee flexion angle at drilling, tunnel position, and tunnel direction; their findings were used for tunnel-geometry inference rather than direct LET clinical outcome inference [28].

#### 3.5.3. Strategies to Reduce Convergence

Jung et al. confirmed in a three-dimensional simulation study that specific combinations of knee flexion angle and tunnel trajectory reduce collision risk in combined ACL and ALL reconstruction [32]. Wasdev et al. described a landmark-guided technique using reproducible surface references for LET femoral entry point selection to reduce convergence risk with the ACL tunnel [41]. Wang et al. identified the anterior cartilage edge of the lateral femoral condyle medial wall as a useful intraoperative reference for avoiding convergence in ACLR with LET [29]. Forsythe et al. showed that femoral fixation point selection also affects isometry in anterolateral reconstruction, although this was used here only as anatomical and kinematic context [33]. Hopper et al. described a technical approach specifically aimed at avoiding knee tunnel convergence when performing a modified Lemaire tenodesis [36].

#### 3.5.4. Intraoperative Prevention Strategies

Preoperative planning reduces but does not eliminate intraoperative convergence risk, as small deviations in entry point or trajectory during drilling can shift an acceptable construct into a collision scenario. Arthroscopic visualization of the ACL femoral tunnel during LET femoral socket drilling provides direct intraoperative feedback, enabling the surgeon to detect an impending tunnel wall breach before it occurs and to adjust trajectory or depth accordingly [35]. This technique applies to anatomic single-bundle ACLR combined with modified Lemaire tenodesis and requires no additional equipment beyond the standard arthroscopic access already in use. Intraoperative tunnel visualization therefore complements preoperative planning as a practical, cost-neutral strategy for combined procedures.

## 4. Discussion

The principal finding of this review is that femoral fixation in LET should not be regarded as a minor technical detail but as a distinct variable that may influence construct behavior, tunnel safety, and, potentially, clinical outcomes. Across cadaveric, imaging, and clinical domains, the fixation family, implant geometry, tunnel trajectory, and knee flexion angle at fixation emerged as recurrent determinants of restraint behavior and tunnel interaction. The clinical relevance of this observation is twofold: first, it reframes fixation selection as an active planning decision rather than a default step; second, it identifies femoral tunnel convergence and collision as modifiable, largely preventable technical risks. To organize this evidence, we propose a fixation taxonomy and a collision-aware planning framework that translate heterogeneous findings into a structured description of the available options and their principal trade-offs.

### 4.1. Fixation Selection and the Collision-Aware Planning Framework

#### 4.1.1. When to Consider Each Fixation Family

Fixation selection should be driven first by tunnel burden and convergence or collision risk, then by revision constraints, implant profile, and fixation-angle control [14,15,16,17]. Inlay interference screw fixation may be considered when a dedicated LET tunnel is feasible, the corridor is unconstrained, and convergence risk remains acceptable after trajectory optimization [9,10]; however, it should not be interpreted as the default option whenever the corridor appears adequate. Onlay fixation is attractive when tunnel burden must be minimized, particularly in revision settings, with prior tunnels, or within constrained lateral femoral corridors; anchor and all-suture anchor constructs provide low-profile alternatives with biomechanical, small-series, and limited comparative evidence [21,22,23,26]. Staple fixation remains a simple option but carries documented ACL tunnel penetration and hardware irritation or removal concerns [24,44]. Suspensory and indirect concepts may be considered when no additional femoral implant is desired, particularly when the ACL already uses a button-based construct [38,39], although independent validation remains limited. Across all families, the fixation angle and tensioning method must be standardized and reported [12,13].

#### 4.1.2. The Planning Framework

**Figure 3:**
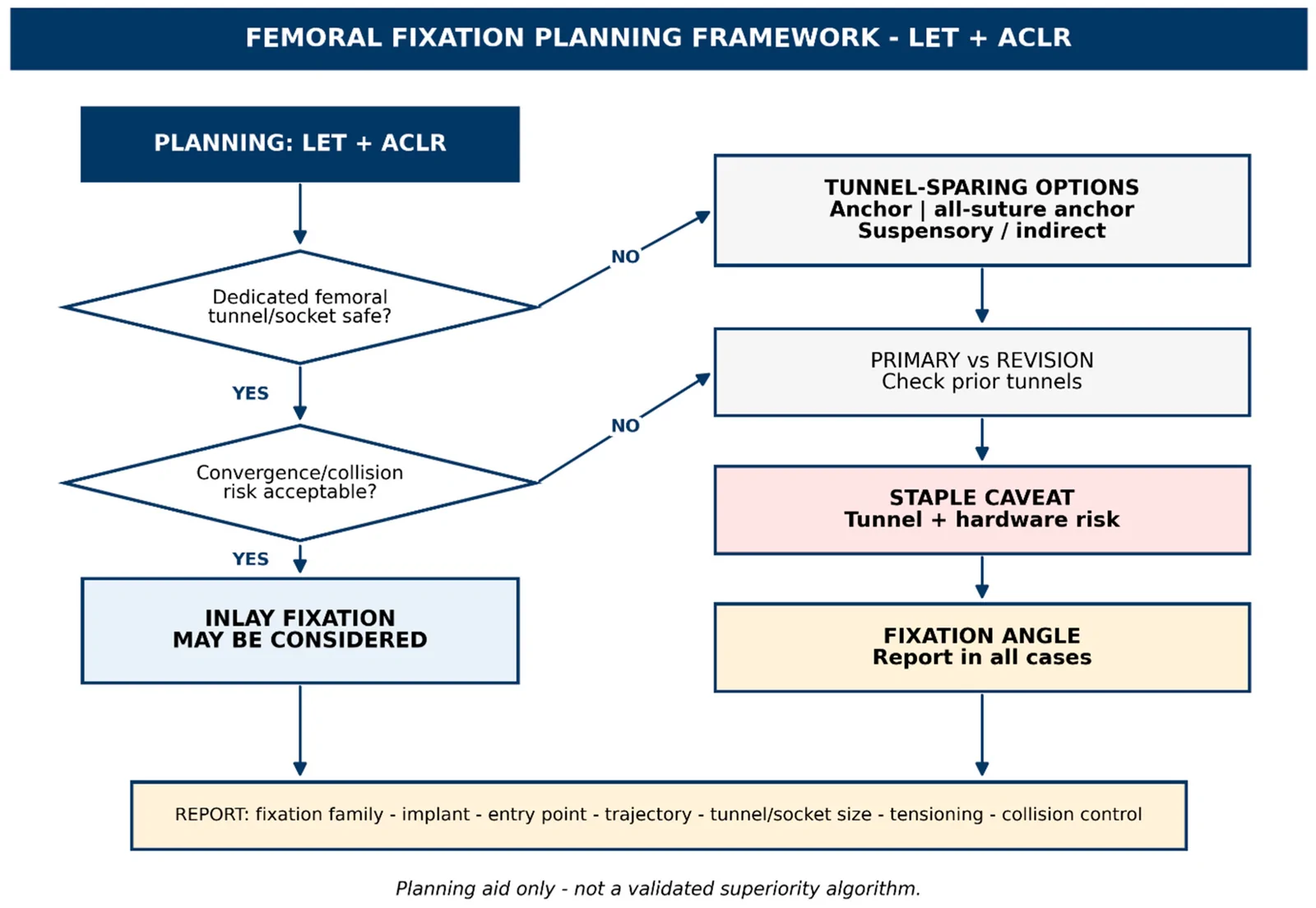
Collision-aware planning framework for femoral fixation family selection in LET combined with ACL reconstruction. The framework is intended as an evidence-informed planning aid, not a validated clinical decision-making instrument or superiority ranking. It separates inlay fixation from tunnel-sparing/tunnel-independent options, highlights primary versus revision considerations, and adds mandatory reporting of fixation angle and tunnel-collision control. (ACL, anterior cruciate ligament; ACLR, ACL reconstruction; LET, lateral extra-articular tenodesis).

Figure 3 presents these principles as an evidence-informed planning framework organized around tunnel burden, corridor safety, trajectory optimization, primary versus revision context, implant profile, and the mandatory reporting of the fixation angle. The framework is not a validated clinical decision-making instrument and should not be interpreted as a superiority ranking, as no fixation family has demonstrated superior clinical outcomes over another in direct comparative trials. All branches share the same documentation requirement: fixation family, implant type, femoral entry point, tunnel or implant trajectory, tunnel or socket dimensions when applicable, graft tensioning method, knee flexion angle during drilling and fixation, and collision-control strategy.

### 4.2. Comparison with Existing Literature

The findings of this review are consistent with, and extend, previous syntheses of lateral extra-articular procedures. Prior reviews have confirmed the clinical benefit of adding LET to ACLR in high-risk patients and have documented wide variability in technique and reporting [4,19,20], but they have generally treated LET as a single entity and have not systematically dissected the femoral fixation step. Similarly, meta-analyses focused on the knee flexion angle at fixation and on graft-failure prevention have highlighted inconsistent reporting of fixation parameters [13], and revision-focused series have shown that extra-articular augmentation improves rotational control while leaving the optimal fixation construct undefined [3]. By combining biomechanical, imaging, and clinical evidence around the fixation construct itself, and by explicitly incorporating tunnel convergence and collision, the present review complements this literature and reframes femoral fixation as a comparably important, and currently under-reported, technical variable.

### 4.3. Knowledge Gaps and Future Directions

Fixation-specific clinical interpretation is limited by inconsistent reporting and study heterogeneity. Many clinical series do not report the femoral fixation family, femoral entry point, drilling direction, or fixation angle with sufficient detail for meaningful comparison [13,20]. Fixation-focused synthesis suggests that outcomes may vary across fixation methods, but heterogeneity in technique and indications restricts inference on superiority [19]. Tunnel convergence outcomes are not standardized: the definitions of convergence, clinically relevant bone-bridge thresholds, and the reporting of tunnel trajectories remain variable, limiting translation into uniform operative guidance [16,17,29]. Tunnel-sparing strategies such as indirect or shared suspensory concepts remain incompletely validated with respect to independent tensioning and construct coupling. Future studies should adopt standardized reporting items, including the fixation family, implant or vendor if available, femoral entry point description, drilling direction, tunnel or socket diameter and depth, knee flexion angle during drilling, knee flexion angle during graft fixation, graft tensioning method, and an explicit convergence or collision-control strategy. Prospective comparative studies designed around fixation families, rather than around LET as a single entity, are needed to determine whether fixation strategy influences failure, complications, and functional outcomes (Table S5).

### 4.4. Strengths and Limitations

This review has several strengths. First, it addresses a specific and clinically relevant technical gap: femoral fixation strategy in LET is the most variable component of a widely performed procedure, yet no prior review has systematically mapped fixation families, their biomechanical behavior, and their relationship to tunnel convergence within a single framework. Second, the structured fixation taxonomy introduced here provides an organizing principle that links technique selection to convergence risk, offering a practical scaffold for surgical planning and future comparative research. Third, the proposed planning framework integrates evidence from cadaveric, imaging, and clinical domains into an actionable planning aid; it is explicitly presented as an evidence-informed framework rather than a validated clinical decision tool.

Several limitations should be acknowledged. This review synthesizes heterogeneous evidence, including cadaveric and robotic studies, CT and three-dimensional imaging analyses, technical reports, and a limited body of fixation-oriented clinical evidence. Because outcomes, denominators, fixation families, and measurement methods were inconsistently reported, and because study designs, fixation methods, and reporting varied substantially, quantitative pooling was not feasible for any outcome and the synthesis was necessarily narrative. Some convergence studies involve anterolateral ligament tunnels rather than LET; these were included only when their tunnel-geometry principles were directly applicable to combined ACL plus lateral augmentation planning and were not used to infer clinical superiority of LET fixation strategies. Finally, publication bias toward novel techniques and underreporting of adverse technical events may also influence the available evidence. These limitations constrain the strength of any inference and should be borne in mind when interpreting the conclusions below.

## 5. Conclusions

Within the limits of a narrative synthesis of heterogeneous studies, this review suggests that femoral fixation in LET may be a more important technical variable than is often assumed. The available biomechanical and imaging evidence indicates that the fixation family, implant geometry, tunnel trajectory, and graft fixation angle can influence construct behavior and tunnel safety, but direct comparative clinical evidence remains limited and does not support ranking one fixation family above another. Femoral tunnel convergence and collision appear to be largely modifiable and therefore warrant careful preoperative and intraoperative planning. The fixation taxonomy and collision-aware planning framework presented here are offered as a structured, evidence-informed aid for describing and planning femoral fixation strategies in LET combined with ACLR, not as a validated decision instrument. Adequately powered prospective comparative studies, designed around fixation families and reporting the femoral entry point, drilling trajectory, tunnel or socket dimensions, fixation method, fixation angle, and collision-control strategy in a standardized manner, are needed before firmer recommendations can be made.

## Figures and Tables

**Table 1:** Characteristics and key findings of the 33 included studies, grouped by evidence role.

Study [Ref]	Design (LOE)	Fixation Family/Method	Sample	Key Findings
Clinical evidence				
Johnson [19]	Systematic review and meta-analysis (III)	Mixed families (staple, anchor, suspensory, screw)	17 studies; 2527 patients	ACLR + LET associated with lower graft failure than isolated ACLR; fixation-specific comparisons remained heterogeneous.
Zabrzynski [20]	Systematic review (IV)	Mixed (sutures, staples, anchors, screws, K-wire)	35 studies; 6253 patients	Wide variability in fixation methods; sutures most frequent, then staples, anchors, and screws; limits direct comparison.
Behrendt [21]	Comparative cohort (III)	Onlay suture anchor vs. transosseous	52 patients (revision)	Onlay anchor and transosseous fixation gave comparable short-term outcomes in revision ACLR with LET.
Conteduca [22]	Retrospective case series (IV)	Onlay knotless all-suture (soft) anchor	15 patients (revision)	Soft-anchor fixation gave favorable 2-year outcomes without major complications.
Erdmann [23]	Prospective case series (IV)	Onlay self-punching all-suture anchor	32 patients	Mini-open modified Lemaire LET with all-suture anchor improved clinical scores without early complications.
Cadaveric and biomechanical evidence				
Gilat [9]	Cadaveric biomechanical	Inlay interference screw; onlay anchor; onlay staple	24 knees	Interference screw showed the highest failure load; staple fixation performed least favorably.
Tollefson [10]	Cadaveric biomechanical	Inlay screw; onlay staple; onlay anchor	18 knees	All constructs biomechanically acceptable; interference screw strongest, anchor weakest.
Xu [11]	Cadaveric biomechanical	Cortical surface vs. intra-tunnel	8 knees	Cortical surface fixation produced less overconstraint in deep flexion than intra-tunnel fixation.
Inderhaug [12] *	Cadaveric biomechanical	Anterolateral procedure; fixation angle varied	12 knees	Restraint behavior was angle dependent; flexion angle at fixation is part of the fixation strategy.
Moran [24]	Cadaveric	Onlay staple	20 paired knees	Staple fixation did not eliminate tunnel risk; ACL tunnel violation in 40% of specimens.
Devitt [25] *	Robotic cadaveric	Distal fixation; no proximal femoral implant	12 knees	Distal fixation restored near-native kinematics while avoiding a proximal femoral tunnel.
Tejada [26]	Cadaveric biomechanical	Onlay all-suture anchor	6 knees	All-suture anchor provided sufficient stability as a low-profile onlay option.
Zhu [27]	Cadaveric	Tunnel-based; 12 drilling-angle combinations	12 knees	Collision risk depended mainly on axial tunnel orientation; more anterior drilling reduced conflict.
CT imaging, convergence, and positioning studies				
Kittl [28] *	Cadaveric and imaging	Anterolateral fixation at three femoral positions	10 knees	Convergence strongly influenced by tunnel position, direction, and ACL drilling angle.
Jaecker [14]	Cadaveric	Lemaire vs. MacIntosh positions	10 knees	Lemaire femoral position showed frequent convergence; MacIntosh position did not.
Smeets [15] *	Cadaveric	ALL fixation; standard vs. proximal-anterior	15 knees	A more proximal, anterior trajectory significantly reduced femoral tunnel convergence.
Perelli [16]	CT imaging	Modified Lemaire; 30° proximal/30° anterior	52 patients	Anterior inclination reduced collision risk; angles >20° appeared safest.
Suh [17] *	3D CT analysis (III)	ALL fixation; transverse vs. distal-anterior	227 patients	Distal-anterior orientation reduced convergence compared with a transverse direction.
Wang [29]	CT imaging	Landmark-guided exit point	70 knees	A landmark-guided femoral exit point helped avoid ACL–LET tunnel convergence.
Kawanishi [30] *	CT and computational	ALL fixation; 0° vs. 30° coronal	9 knees	Safety depended on combined ACL/ALL orientation; only limited angle combinations were safe.
Kanakamedala [31]	Clinical imaging (IV)	Landmark/tactile placement, no fluoroscopy	47 patients	Landmark placement was inconsistent; ~half of fixation points outside the isometric zone.
Jung [32] *	3D simulation	ALL femoral tunnel orientation	10 patients	Collision risk minimized by specific flexion-angle and tunnel-orientation combinations.
Forsythe [33] *	Imaging and isometry	Proximal fixation anterior to lateral epicondyle	6 knees	Fixation anterior to the lateral epicondyle improved isometry.
Jaecker [34]	Radiographic and anatomic	Radiographic landmark guidance	10 knees	Radiographic landmarks improved reproducibility of femoral tunnel positioning.
Technical reports and notes				
Mitrousias [35]	Technical note	Inlay screw + arthroscopic tunnel visualization	N/A	Arthroscopic visualization may improve intraoperative detection of an impending tunnel breach.
Hopper [36]	Technical report	Onlay knotless suture anchor (2.6 mm)	N/A	Low-profile onlay anchor proposed as a tunnel-sparing option with reduced hardware prominence.
Capella [37]	Technical report	Cortical suspensory button + suture tape	N/A	Suspensory fixation with suture tape allows controlled tensioning with limited hardware.
Bechis [38]	Technical report	Indirect suspensory via ACL femoral device	N/A	Indirect suspensory fixation avoids a separate LET femoral implant or tunnel.
Koukoulias [39]	Technical report	Shared/common suspensory fixation	N/A	Shared suspensory fixation avoids an additional implant and simplifies the construct.
Manze [40]	Technical report	Single femoral tunnel; over-the-loop	N/A	A single femoral tunnel avoids convergence while reducing implant use and complexity.
Wasdev [41]	Technical note	Landmark-guided tunnel-based placement	N/A	Landmark-guided anterior/proximal pin direction with arthroscopic control reduces convergence risk.
Wait [42]	Technical report	Onlay options (anchor, staple, screw)	N/A	Reviews the practical strengths and limitations of anchor, staple, and screw onlay fixation.
Haus [43]	Technical report	Inlay all-suture knotless anchor	N/A	All-suture knotless anchor presented as a low-profile inlay option for primary and revision settings.

Studies marked with an asterisk (*) are anterolateral ligament (ALL) reconstruction or non–modified-Lemaire lateral augmentation studies included only for tunnel-geometry or biomechanical extrapolation, not as direct modified-Lemaire LET clinical evidence. LOE, level of evidence (Oxford Centre for Evidence-Based Medicine); N/A, not applicable.

**Table 2:** Methodological quality of the included studies appraised with the Methodological Index for Non-Randomized Studies (MINORS).

Study [Ref]	Design	MINORS Applicability (Ideal)	MINORS Score	Comment
Clinical evidence				
Johnson [19]	Systematic review and meta-analysis	Not applicable	—	Evidence synthesis; MINORS not applicable.
Zabrzynski [20]	Systematic review	Not applicable	—	Evidence synthesis; MINORS not applicable.
Behrendt [21]	Comparative cohort	Comparative (24)	19	Two fixation groups with clinical follow-up; comparative cohort without a reported prospective sample-size calculation.
Conteduca [22]	Retrospective case series	Non-comparative (16)	11	Single-arm retrospective case series without a comparator group.
Erdmann [23]	Prospective case series	Non-comparative (16)	12	Single-arm prospective case series without a comparator group.
Cadaveric and biomechanical evidence				
Gilat [9]	Cadaveric biomechanical	Non-comparative (16)	13	Controlled construct comparison; objective load-to-failure endpoint.
Tollefson [10]	Cadaveric biomechanical	Non-comparative (16)	13	Controlled construct comparison; objective failure-load endpoint.
Xu [11]	Cadaveric biomechanical	Non-comparative (16)	12	Paired construct comparison; small specimen number.
Inderhaug [12] *	Cadaveric biomechanical	Non-comparative (16)	13	Robotic testing across fixation angles; objective kinematic endpoint.
Moran [24]	Cadaveric	Non-comparative (16)	12	Paired design; objective tunnel-violation endpoint.
Devitt [25] *	Robotic cadaveric	Non-comparative (16)	13	Robotic kinematic testing; near-native comparison.
Tejada [26]	Cadaveric biomechanical	Non-comparative (16)	11	Small specimen number; single-construct stability testing.
Zhu [27]	Cadaveric	Non-comparative (16)	12	Multiple drilling-angle combinations; objective collision endpoint.
CT imaging, convergence, and positioning studies				
Kittl [28] *	Cadaveric and imaging	Non-comparative (16)	12	Objective convergence endpoint across positions.
Jaecker [14]	Cadaveric	Non-comparative (16)	12	Position comparison; objective convergence endpoint.
Smeets [15] *	Cadaveric	Non-comparative (16)	12	Trajectory comparison; objective convergence endpoint.
Perelli [16]	CT imaging	Non-comparative (16)	13	In vivo CT; objective angle-dependent collision endpoint.
Suh [17] *	3D CT analysis	Non-comparative (16)	13	Large 3D CT cohort; objective convergence endpoint.
Wang [29]	CT imaging	Non-comparative (16)	12	Landmark-definition study; objective measurement endpoint.
Kawanishi [30] *	CT and computational	Non-comparative (16)	12	Computational orientation analysis; small specimen number.
Kanakamedala [31]	Clinical imaging	Non-comparative (16)	12	In vivo positioning study; non-comparative imaging analysis.
Jung [32] *	3D simulation	Non-comparative (16)	12	Simulation study; objective collision endpoint.
Forsythe [33] *	Imaging and isometry	Non-comparative (16)	11	Small specimen number; isometry measurement.
Jaecker [34]	Radiographic and anatomic	Non-comparative (16)	12	Reproducibility study; objective landmark endpoint.
Technical reports and notes				
Mitrousias [35]	Technical note	Not applicable	—	Descriptive technical note; MINORS not applicable, appraised narratively.
Hopper [36]	Technical report	Not applicable	—	
Capella [37]	Technical report	Not applicable	—	
Bechis [38]	Technical report	Not applicable	—	
Koukoulias [39]	Technical report	Not applicable	—	
Manze [40]	Technical report	Not applicable	—	
Wasdev [41]	Technical note	Not applicable	—	
Wait [42]	Technical report	Not applicable	—	
Haus [43]	Technical report	Not applicable	—	

MINORS global scores were assigned from the methodological descriptions in each published report. Non-comparative studies are scored out of 16 and comparative studies out of 24. Systematic reviews and purely descriptive technical notes are not amenable to MINORS and are appraised with the noted alternative approach. * ALL/non–modified-Lemaire studies used for extrapolation only.

## Data Availability

The data supporting this review are available within the article and the Supplementary Materials. Table 1 provides the evidence table of the included studies, and Table 2 provides the methodological quality (MINORS) appraisal. Supplementary Table S1 provides the completed PRISMA 2020 checklist, and Supplementary Table S2 provides the full electronic search strategies; Supplementary Table S3 provides the detailed evidence table of the included studies and their role in the synthesis; Supplementary Table S4 summarizes the evidence on tunnel convergence/collision and its application to LET planning; and Supplementary Table S5 provides the proposed minimum reporting checklist for LET femoral fixation studies. The study screening database (study map) is available from the corresponding author upon reasonable request.

## Supplementary Materials

The following supporting information can be downloaded at: https://www.mdpi.com/article/10.3390/medicina62081542/s1, Table S1: PRISMA 2020 checklist; Table S2: Electronic search strategies; Table S3: Detailed evidence table of the included studies and their role in the synthesis; Table S4: Tunnel convergence/collision evidence and application to LET planning; Table S5: Proposed minimum reporting checklist for LET femoral fixation studies.

## Abbreviations

The following abbreviations are used in this manuscript:

ACL	Anterior cruciate ligament
ACLR	Anterior cruciate ligament reconstruction
ALL	Anterolateral ligament
CT	Computed tomography
ITB	Iliotibial band
LET	Lateral extra-articular tenodesis
OCEBM	Oxford Centre for Evidence-Based Medicine
PRISMA	Preferred Reporting Items for Systematic Reviews and Meta-Analyses
