# Supplementary material for: Femoral Fixation Techniques for Lateral Extra-Articular Tenodesis During Anterior Cruciate Ligament Reconstruction: A Systematic Review and Proposed Collision-Aware Planning Framework
Source: Medicina (Kaunas). 2026 Aug 11;62(8):1542. doi: 10.3390/medicina62081542 (PMC13515370; doi:10.3390/medicina62081542)
Supplement: Supplementary file 1 [file medicina-62-01542-s001.zip › medicina-4337588-supplementary.pdf]

Supplementary Materials

Femoral Fixation Techniques for Lateral Extra-Articular Tenodesis During Anterior Cruciate Ligament Reconstruction: A Systematic Review and Proposed Collision-Aware Planning Framework

Table S1. PRISMA 2020 checklist.

Section and topic	Item	Checklist item	Location where item is reported
TITLE — Title	1	Identify the report as a systematic review.	Title page
ABSTRACT — Abstract	2	See the PRISMA 2020 for Abstracts checklist.	Abstract
INTRODUCTION — Rationale	3	Describe the rationale for the review in the context of existing knowledge.	Section 1
INTRODUCTION — Objectives	4	Provide an explicit statement of the objective(s) or question(s) the review addresses.	Section 1
METHODS — Eligibility criteria	5	Specify the inclusion and exclusion criteria for the review and how studies were grouped for the syntheses.	Section 2.3
METHODS — Information sources	6	Specify all databases, registers, websites, organisations, reference lists and other sources searched or consulted to identify studies. Specify the date when each source was last searched or consulted.	Section 2.2; Table S2
METHODS — Search strategy	7	Present the full search strategies for all databases, registers and websites, including any filters and limits used.	Section 2.2; Table S2
METHODS — Selection process	8	Specify the methods used to decide whether a study met the inclusion criteria of the review, including how many reviewers screened each record and each report retrieved, whether they worked independently, and, if applicable, details of automation tools used in the process.	Sections 2.3 and 3.1; Figure 1
METHODS — Data collection process	9	Specify the methods used to collect data from reports, including how many reviewers collected data from each report, whether they worked independently, any processes for obtaining or confirming data from study investigators, and, if applicable, details of automation tools used in the process.	Section 2.4; reviewer details not reported
METHODS — Data items	10a	List and define all outcomes for which data were sought. Specify whether all results compatible with each outcome domain in each study were sought and, if not, the methods used to decide which results to collect.	Sections 2.3–2.4
METHODS — Data items	10b	List and define all other variables for which data were sought (for example, participant and intervention characteristics and funding sources). Describe any assumptions made about missing or unclear information.	Section 2.4; Table 1; Table S3
METHODS — Study risk-of-bias assessment	11	Specify the methods used to assess risk of bias in the included studies, including details of the tool(s) used, how many reviewers assessed each study and whether they worked independently, and, if applicable, details of automation tools used in the process.	Section 2.5; Table 2
METHODS — Effect measures	12	Specify for each outcome the effect measure(s) used in the synthesis or presentation of results.	Section 2.6; no meta-analysis
METHODS — Synthesis methods	13a	Describe the processes used to decide which studies were eligible for each synthesis, such as tabulating study intervention characteristics and comparing them with the planned groups for each synthesis.	Sections 2.3–2.4
METHODS — Synthesis methods	13b	Describe any methods required to prepare the data for presentation or synthesis, such as handling missing summary statistics or data conversions.	Section 2.6; no quantitative conversion
METHODS — Synthesis methods	13c	Describe any methods used to tabulate or visually display results of individual studies and syntheses.	Section 2.4; Table 1; Tables S3–S4
METHODS — Synthesis methods	13d	Describe any methods used to synthesize results and provide a rationale for the choice(s). If meta-analysis was performed, describe the model(s), statistical heterogeneity methods, and software used.	Section 2.6
METHODS — Synthesis methods	13e	Describe any methods used to explore possible causes of heterogeneity among study results, such as subgroup analysis or meta-regression.	Section 2.6; no formal subgroup analysis
METHODS — Synthesis methods	13f	Describe any sensitivity analyses conducted to assess robustness of the synthesized results.	Not applicable; narrative synthesis
METHODS — Reporting-bias assessment	14	Describe any methods used to assess risk of bias due to missing results in a synthesis (arising from reporting biases).	Not formally assessed; Section 4.4

Section and topic	Item	Checklist item	Location where item is reported
METHODS — Certainty assessment	15	Describe any methods used to assess certainty (or confidence) in the body of evidence for an outcome.	Section 2.5; Table 2; no formal GRADE
RESULTS — Study selection	16a	Describe the results of the search and selection process, from the number of records identified to the number of studies included, ideally using a flow diagram.	Section 3.1; Figure 1
RESULTS — Study selection	16b	Cite studies that might appear to meet the inclusion criteria but were excluded, and explain why they were excluded.	Section 3.1; grouped reasons only
RESULTS — Study characteristics	17	Cite each included study and present its characteristics.	Table 1; Table S3
RESULTS — Risk of bias in studies	18	Present assessments of risk of bias for each included study.	Table 2
RESULTS — Results of individual studies	19	For all outcomes, present for each study: (a) summary statistics for each group, where appropriate, and (b) an effect estimate and its precision, ideally using structured tables or plots.	Sections 3.2–3.5; Table 1; Table S3
RESULTS — Results of syntheses	20a	For each synthesis, briefly summarise the characteristics and risk of bias among contributing studies.	Sections 3.2–3.5; Tables 1–2
RESULTS — Results of syntheses	20b	Present results of all statistical syntheses conducted. If meta-analysis was done, present the summary estimate, its precision, and measures of statistical heterogeneity.	Not applicable; narrative synthesis
RESULTS — Results of syntheses	20c	Present results of all investigations of possible causes of heterogeneity among study results.	Not applicable; no formal investigation
RESULTS — Results of syntheses	20d	Present results of all sensitivity analyses conducted to assess robustness of the synthesized results.	Not applicable; no sensitivity analysis
RESULTS — Reporting biases	21	Present assessments of risk of bias due to missing results (arising from reporting biases) for each synthesis assessed.	Not formally assessed; Section 4.4
RESULTS — Certainty of evidence	22	Present assessments of certainty (or confidence) in the body of evidence for each outcome assessed.	Section 2.5; Table 2; no formal GRADE
DISCUSSION — Discussion	23a	Provide a general interpretation of the results in the context of other evidence.	Sections 4.1–4.3
DISCUSSION — Discussion	23b	Discuss any limitations of the evidence included in the review.	Section 4.4
DISCUSSION — Discussion	23c	Discuss any limitations of the review processes used.	Section 4.4
DISCUSSION — Discussion	23d	Discuss implications of the results for practice, policy, and future research.	Sections 4.1–4.3 and 5
OTHER INFORMATION — Registration and protocol	24a	Provide registration information for the review, including register name and registration number, or state that the review was not registered.	Section 2.1; PROSPERO CRD420261396698
OTHER INFORMATION — Registration and protocol	24b	Indicate where the review protocol can be accessed, or state that a protocol was not prepared.	Section 2.1; PROSPERO record
OTHER INFORMATION — Registration and protocol	24c	Describe and explain any amendments to information provided at registration or in the protocol.	No amendments reported
OTHER INFORMATION — Support	25	Describe sources of financial or non-financial support for the review and the role of funders or sponsors.	Funding statement
OTHER INFORMATION — Competing interests	26	Declare any competing interests of review authors.	Conflicts of Interest statement
OTHER INFORMATION — Availability of data, code and other materials	27	Report which review materials are publicly available and where they can be found, including data collection forms, extracted data, analysis data, analytic code, and other materials.	Data Availability Statement; Supplementary Materials

Source: Page MJ, McKenzie JE, Bossuyt PM, et al. The PRISMA 2020 statement: an updated guideline for reporting systematic reviews. BMJ. 2021;372:n71. doi:10.1136/bmj.n71. Checklist text adapted under CC BY 4.0.

Table S2. Electronic search strategies.

Source	Search date	Limits	Records	Exact search strategy or method
PubMed/MEDLINE	February 2026	English language; inception to February 2026	324	(("lateral extra-articular tenodesis" OR LET OR "modified Lemaire" OR Lemaire OR MacIntosh OR "Coker-Arnold" OR "anterolateral ligament" OR "anterolateral augmentation" OR ALL) AND ("anterior cruciate ligament reconstruction" OR "ACL reconstruction" OR ACLR) AND ("femoral fixation" OR fixation OR tunnel OR socket OR "tunnel convergence" OR "tunnel collision" OR biomechanical OR cadaveric OR CT OR "computed tomography"))
Embase	February 2026	English language; inception to February 2026	353	(('lateral extra-articular tenodesis',ab,kw OR LET,ab,kw OR 'modified Lemaire',ab,kw OR Lemaire,ab,kw OR MacIntosh,ab,kw OR 'Coker-Arnold',ab,kw OR 'anterolateral ligament',ab,kw OR 'anterolateral augmentation',ab,kw OR ALL,ab,kw) AND ('anterior cruciate ligament reconstruction',ab,kw OR 'ACL reconstruction',ab,kw OR ACLR,ab,kw) AND ('femoral fixation',ab,kw OR fixation,ab,kw OR tunnel,ab,kw OR socket,ab,kw OR 'tunnel convergence',ab,kw OR 'tunnel collision',ab,kw OR biomechanical,ab,kw OR cadaveric,ab,kw OR CT,ab,kw OR 'computed tomography',ab,kw))
Cochrane Library	February 2026	Trials/reviews; inception to February 2026	94	#1 ("lateral extra-articular tenodesis" OR LET OR "modified Lemaire" OR Lemaire OR MacIntosh OR "Coker-Arnold" OR "anterolateral ligament" OR "anterolateral augmentation" OR ALL),ab,kw #2 ("anterior cruciate ligament reconstruction" OR "ACL reconstruction" OR ACLR),ab,kw #3 ("femoral fixation" OR fixation OR tunnel OR socket OR "tunnel convergence" OR "tunnel collision" OR biomechanical OR cadaveric OR CT OR "computed tomography"),ab,kw #4 #1 AND #2 AND #3
Other sources	February 2026	Reference-list searching of included studies and relevant reviews	43	Reference lists of included studies and relevant reviews were searched manually; all records were imported into the same screening database before deduplication.

The database searches identified 771 records (PubMed/MEDLINE, n = 324; Embase, n = 353; Cochrane Library, n = 94); 43 additional records were identified through reference-list searching. Searches were conducted through February 2026. Abbreviations: ACLR, anterior cruciate ligament reconstruction; ALL, anterolateral ligament; CT, computed tomography; LET, lateral extra-articular tenodesis.

Table S3. Detailed evidence table of the included studies and their role in the synthesis.

First author [reference]	Study design	LOE	LET procedure	Study sample or unit	Femoral fixation family or method	Key finding	Evidence role	Use in synthesis
**Clinical evidence**								
Johnson 2026 [37]	Systematic review and meta-analysis	III	LET + ACLR (all types)	17 studies; 2,527 patients	Mixed fixation families, staple, suture anchor, cortical suspensory button, interference screw	ACLR + LET was associated with lower graft failure than isolated ACLR, although fixation-specific comparisons remained heterogeneous.	Direct LET clinical	Clinical inference; limited by heterogeneity/sample size
Zabrzynski 2025 [38]	Systematic review	IV	LET + ACLR (modified Lemaire, MacIntosh, Coker-Arnold)	35 studies; 6,253 patients	Mixed fixation methods, sutures, staples, suture anchors, interference screws, K-wire, bioabsorbable screws, titanium screws	Femoral fixation methods varied substantially across studies, with sutures reported most often, followed by staples, anchors, and screws.	Direct LET technical/clinical synthesis	Technical heterogeneity and clinical context
Behrendt 2023 [22]	Comparative cohort study	III	LET in revision ACLR	52 patients	Onlay suture anchor vs transosseous fixation	Onlay suture anchor and transosseous fixation showed comparable short-term outcomes in revision ACLR with LET.	Direct LET clinical	Clinical inference; limited by heterogeneity/sample size
Conteduca 2025 [24]	Retrospective case series	IV	Combined revision ACLR + modified Lemaire LET	15 patients	Onlay knotless all-suture anchor (soft anchor)	Soft-anchor fixation yielded favorable 2-year outcomes without major complications in revision ACLR with modified Lemaire LET.	Direct LET clinical	Clinical inference; limited by heterogeneity/sample size
Erdmann 2025 [25]	Prospective case series	IV	Primary and revision ACLR + modified Lemaire LET, mini-open	32 patients	Onlay self-punching all-suture anchor	Mini-open modified Lemaire LET with all-suture anchor fixation improved clinical scores without early complications.	Direct LET clinical	Clinical inference; limited by heterogeneity/sample size
**Cadaveric and biomechanical evidence**								
Gilat 2025 [9]	Cadaveric biomechanical study	N/A	Modified Lemaire LET	24 cadaveric knees	Inlay interference screw, onlay suture anchor, onlay staple	Interference screw fixation showed the highest failure load, whereas staple fixation performed least favorably.	LET biomechanical or cadaveric	Biomechanical/technical inference
Tollefson 2025 [10]	Cadaveric biomechanical study	N/A	Modified Lemaire LET	18 cadaveric knees	Inlay interference screw, onlay staple, onlay suture anchor	All fixation types were biomechanically acceptable, but interference screw fixation was strongest and anchor fixation was weakest.	LET biomechanical or cadaveric	Biomechanical/technical inference
Xu 2022 [11]	Cadaveric biomechanical study	N/A	Modified Lemaire LET	8 cadaveric knees	Cortical surface fixation vs intra-tunnel fixation	Cortical surface fixation produced less overconstraint in deep flexion than intra-tunnel fixation.	LET biomechanical or cadaveric	Biomechanical/technical inference
Inderhaug 2017 [12]*	Cadaveric biomechanical study	N/A	Modified Lemaire LET vs ALLR	12 cadaveric knees	Anterolateral procedure; fixation angle varied	Modified Lemaire LET restored native-like kinematics across fixation angles, although restraint behavior remained angle dependent.	LET biomechanical or cadaveric	Biomechanical/technical inference
Moran 2023 [20]	Cadaveric study	N/A	ACLR + LET, staple fixation	20 paired cadaveric knees	Onlay staple	Staple fixation did not eliminate tunnel risk, with ACL tunnel violation observed in 40% of specimens.	LET biomechanical or cadaveric	Biomechanical/technical inference
Devitt 2019 [32]*	Robotic cadaveric study	N/A	Distally fixed lateral augmentation (modified Ellison)	12 cadaveric knees	Distal fixation; no proximal femoral implant	Distal fixation restored near-native kinematics while avoiding a proximal femoral tunnel.	LET biomechanical or cadaveric	Biomechanical/technical inference
Tejada 2025 [23]	Cadaveric biomechanical study	N/A	Modified Lemaire LET	6 cadaveric knees	Onlay all-suture anchor	All-suture anchor fixation provided sufficient biomechanical stability as a low-profile LET option.	LET biomechanical or cadaveric	Biomechanical/technical inference
Zhu 2021 [33]	Cadaveric study	N/A	Modified Lemaire LET + ACLR	12 cadaveric knees	Tunnel-based LET fixation; 12 drilling-angle combinations	Tunnel collision risk depended mainly on axial tunnel orientation, with more anterior drilling reducing conflict.	LET biomechanical or cadaveric	Biomechanical/technical inference
**CT imaging, convergence, and positioning studies**								
Kittl 2022 [40]*	Cadaveric and imaging study	N/A	ACLR + anterolateral structure reconstruction	10 cadaveric knees	Tunnel-based anterolateral fixation at three femoral positions	Tunnel convergence was strongly influenced by tunnel position, tunnel direction, and ACL drilling angle.	ALL/broader lateral augmentation extrapolation	Tunnel-geometry inference only

First author [reference]	Study design	LOE	LET procedure	Study sample or unit	Femoral fixation family or method	Key finding	Evidence role	Use in synthesis
Jaecker 2019 [14]	Cadaveric study	N/A	Lemaire and MacIntosh LET	10 cadaveric knees	Tunnel-based LET fixation; Lemaire vs MacIntosh positions	The Lemaire femoral position showed frequent tunnel convergence, whereas the MacIntosh position did not.	Direct LET tunnel-safety evidence	Tunnel convergence/collision planning
Smeets 2019 [15]*	Cadaveric study	N/A	ACLR + ALL reconstruction	15 cadaveric knees	Tunnel-based ALL femoral fixation; standard vs proximal-anterior angulation	A more proximal and anterior tunnel trajectory significantly reduced femoral tunnel convergence.	ALL/broader lateral augmentation extrapolation	Tunnel-geometry inference only
Perelli 2020 [16]	CT-based imaging study	N/A	ACLR + modified Lemaire tenodesis	52 patients; CT analysis	Tunnel-based modified Lemaire fixation; 30° proximal and 30° anterior inclination	Anterior tunnel inclination reduced collision risk, with angles greater than 20° appearing safest.	Direct LET tunnel-safety evidence	Tunnel convergence/collision planning
Suh 2024 [17]*	3D CT analysis	III	ACLR + ALLR, distally vs anteriorly directed ALL tunnel	227 patients; 3D CT analysis	Tunnel-based ALL femoral fixation; transverse vs distal-anterior orientation	Distal-anterior tunnel orientation reduced convergence compared with a transverse direction.	ALL/broader lateral augmentation extrapolation	Tunnel-geometry inference only
Wang 2025 [43]	CT-based imaging study	N/A	ACLR + LET, tunnel-based	70 knees total; CT analysis	Tunnel-based LET; landmark-guided exit point	A landmark-guided femoral exit point was identified to help avoid ACL LET tunnel convergence.	Direct LET tunnel-safety evidence	Tunnel convergence/collision planning
Kawanishi 2023 [35]*	CT and computational analysis	N/A	Double-bundle ACLR + ALLR	9 cadaveric knees	Tunnel-based ALL femoral fixation; 0° vs 30° coronal orientation	Tunnel safety depended on the combined orientation of ACL and ALL tunnels, with only limited angle combinations appearing safe.	ALL/broader lateral augmentation extrapolation	Tunnel-geometry inference only
Kanakamedala 2024 [39]	Clinical imaging study	IV	LET + ACLR, landmark-based	47 patients	Tunnel-based LET, landmark- and tactile-guided femoral placement without fluoroscopy	Landmark-based LET femoral placement was inconsistently accurate, with nearly half of fixation points outside the isometric zone.	Direct LET tunnel-safety evidence	Tunnel convergence/collision planning
Jung 2025 [41]*	3D simulation study	N/A	ACLR + ALL reconstruction	10 patients; 3D simulation	ALL femoral tunnel orientation	Tunnel collision risk was minimized by specific combinations of ACL drilling flexion angle and ALL tunnel orientation.	ALL/broader lateral augmentation extrapolation	Tunnel-geometry inference only
Forsythe 2019 [44]*	Imaging and isometry study	N/A	ALL reconstruction	6 cadaveric knees; imaging analysis	Proximal femoral fixation anterior to the lateral epicondyle	Femoral fixation anterior to the lateral epicondyle improved isometry and may optimize graft behavior.	ALL/broader lateral augmentation extrapolation	Tunnel-geometry inference only
Jaecker 2019b [34]	Radiographic and anatomic study	N/A	LET femoral tunnel positioning	10 cadaveric knees	Tunnel-based LET; radiographic landmark guidance	Radiographic landmarks improved the reproducibility of LET femoral tunnel positioning.	Direct LET tunnel-safety evidence	Tunnel convergence/collision planning
**Technical reports and notes**								
Mitrousias 2023 [19]	Technical note	N/A	Anatomic ACLR + modified Lemaire LET	N/A	Inlay interference screw plus arthroscopic tunnel visualization	Arthroscopic tunnel visualization may improve intraoperative detection of impending tunnel breach.	Direct LET tunnel-safety evidence	Tunnel convergence/collision planning
Hopper 2022 [26]	Technical report	N/A	Modified Lemaire LET	N/A	Onlay knotless suture anchor (2.6 mm)	Low-profile onlay anchor fixation was proposed as a tunnel-sparing option with reduced hardware prominence.	Direct LET tunnel-safety evidence	Tunnel convergence/collision planning
Capella 2024 [29]	Technical report	N/A	Modified Lemaire LET with suture tape augmentation	N/A	Cortical suspensory button with suture tape augmentation	Cortical suspensory fixation with suture tape augmentation was proposed to allow controlled tensioning with limited hardware burden.	Technical guidance	Feasibility/operative description; not comparative efficacy
Bechis 2024 [30]	Technical report	N/A	Modified Lemaire LET + ACLR	N/A	Indirect suspensory fixation via ACL femoral device	Indirect suspensory fixation through the ACL device avoided a separate LET femoral implant or tunnel.	Technical guidance	Feasibility/operative description; not comparative efficacy
Koukoulias 2022 [31]	Technical report	N/A	ACLR + modified Lemaire tenodesis	N/A	Shared/common suspensory femoral fixation	Shared suspensory fixation was proposed to avoid an additional femoral implant and simplify construct design.	Technical guidance	Feasibility/operative description; not comparative efficacy
Manze 2025 [36]	Technical report	N/A	ACLR (quadrupled hamstring) + modified Lemaire LET	N/A	Single femoral tunnel; over-the-loop technique	A single femoral tunnel technique was proposed to avoid convergence while reducing implant use and technical complexity.	Technical guidance	Feasibility/operative description; not comparative efficacy

First author [reference]	Study design	LOE	LET procedure	Study sample or unit	Femoral fixation family or method	Key finding	Evidence role	Use in synthesis
Wasdev 2023 [42]	Technical note	N/A	LET + ACLR	N/A	Landmark-guided tunnel-based placement	Landmark-guided anterior and proximal pin direction with arthroscopic control was proposed to reduce convergence risk.	Direct LET tunnel-safety evidence	Tunnel convergence/collision planning
Wait 2023 [28]	Technical report	N/A	ACLR + LET, onlay technique	N/A	Onlay fixation options, anchor, staple, interference screw	This technical review outlined the practical strengths and limitations of anchor, staple, and screw onlay fixation.	Technical guidance	Feasibility/operative description; not comparative efficacy
Haus 2023 [27]	Technical report	N/A	Modified Lemaire LET	N/A	Inlay technique with all-suture knotless anchor fixation	All-suture knotless anchor fixation was presented as a low-profile inlay option for both primary and revision settings.	Technical guidance	Feasibility/operative description; not comparative efficacy

* ALL reconstruction or non-modified-Lemaire lateral augmentation studies were included only for tunnel-geometry or biomechanical extrapolation, not as direct modified-Lemaire LET clinical evidence. Abbreviations: ACLR, anterior cruciate ligament reconstruction; ALL, anterolateral ligament; ALLR, anterolateral ligament reconstruction; CT, computed tomography; LET, lateral extra-articular tenodesis; LOE, level of evidence; N/A, not applicable.

Table S4. Tunnel convergence/collision evidence and application to LET planning.

Study	Procedure or construct	Method or design	Tunnel-interaction assessment	Key finding or technical implication	Application to LET planning
**DIRECT TUNNEL-INTERACTION EVIDENCE**					
Moran 2023 [20]	ACLR + LET with onlay staple fixation	Cadaveric study	Actual ACL femoral-tunnel violation by staple hardware	ACL tunnel violation occurred in 40% of specimens; staple fixation did not eliminate tunnel risk.	Confirm staple position and prong trajectory relative to the ACL femoral tunnel.
Zhu 2021 [33]	Modified Lemaire LET + ACLR; tunnel-based fixation	Cadaveric study; 12 drilling-angle combinations	Observed intersection/collision of LET and ACL femoral tunnels	Collision depended mainly on axial orientation; a more anterior LET tunnel trajectory reduced conflict.	If a tunnel is required, prioritize anterior axial angulation while confirming the ACL tunnel course.
Kittl 2022 [40]*	ACLR + anterolateral reconstruction at three femoral positions	Cadaveric and imaging study	Tunnel convergence across combinations of femoral position, tunnel direction, and ACL drilling angle	Convergence varied markedly with both entry point and drilling trajectory.	Treat entry point and trajectory as linked planning variables; applicability is geometric extrapolation.
Jaecker 2019 [14]	Lemaire and MacIntosh LET	Cadaveric study	Observed convergence between LET and ACL femoral tunnels	Frequent convergence occurred at the Lemaire position; none was observed at the MacIntosh position in the tested configuration.	Choose the fixation position with explicit consideration of the existing/planned ACL tunnel.
Smeets 2019 [15]*	ACLR + ALL reconstruction	Cadaveric study	Observed convergence for standard versus proximal-anterior ALL tunnel trajectories	A more proximal and anterior trajectory significantly reduced convergence.	Use only as geometric extrapolation; anterior/proximal redirection may reduce tunnel conflict.
Perelli 2020 [16]	ACLR + modified Lemaire LET	In vivo CT imaging study	CT-defined ACL–LET tunnel collision according to tunnel inclination	Anterior inclination reduced collision; angles greater than 20° appeared safest in the tested cohort.	For tunnel-based LET, consider an anterior inclination greater than 20° while accounting for patient anatomy.
Suh 2024 [17]*	ACLR + ALL reconstruction	3D CT analysis	3D CT convergence for transverse versus distal-anterior ALL tunnel orientation	Distal-anterior orientation reduced convergence compared with a transverse direction.	Use as geometric extrapolation only; avoid a purely transverse tunnel when paths would overlap.
Wang 2025 [43]	ACLR + tunnel-based LET	CT-based imaging study	CT relationship between the ACL tunnel and a landmark-guided LET exit point	A landmark-guided femoral exit point was identified to reduce convergence risk.	Use reproducible landmarks, but confirm the actual ACL tunnel trajectory intraoperatively.
Kawanishi 2023 [35]*	Double-bundle ACLR + ALL reconstruction	CT and computational analysis	Computed collision across combinations of ACL and ALL tunnel orientation	Only limited angle combinations were collision-free.	Use as geometric extrapolation; assess the combined orientation of all femoral tunnels.
Jung 2025 [41]*	ACLR + ALL reconstruction	3D simulation study	Simulated collision across ACL drilling flexion angles and ALL tunnel orientations	Collision risk was minimized by specific paired angle combinations.	Plan ACL and lateral tunnel trajectories together; applicability is geometric extrapolation.
**INDIRECT POSITIONING OR TUNNEL-SPARING EVIDENCE**					
Xu 2022 [11]	Modified Lemaire LET; cortical surface versus intratunnel fixation	Cadaveric biomechanical study	No direct collision endpoint; compared graft behavior by fixation location	Cortical surface fixation produced less deep-flexion overconstraint than intratunnel fixation.	An onlay option may avoid a separate tunnel while limiting deep-flexion overconstraint.
Devitt 2019 [32]*	Distally fixed lateral augmentation (modified Ellison)	Robotic cadaveric study	No proximal femoral tunnel or implant	Distal fixation restored near-native kinematics while inherently avoiding proximal tunnel conflict.	Consider a distal-fixation construct when avoidance of a femoral tunnel is a priority; not direct modified-Lemaire evidence.
Kanakamedala 2024 [39]	LET + ACLR with landmark/tactile-guided placement	Clinical imaging study	Position relative to the radiographic isometric zone; no direct collision endpoint	Nearly half of fixation points were outside the isometric zone.	Landmarks alone may be insufficient; confirm fixation position and tunnel course with adjunctive visualization when needed.
Forsythe 2019 [44]*	ALL reconstruction	Imaging and isometry study	Isometry by femoral fixation point; no direct collision endpoint	A point anterior to the lateral epicondyle improved isometry in the tested construct.	Use only as anatomic/geometry extrapolation; isometry and tunnel safety must both be considered.
Jaecker 2019b [34]	LET femoral tunnel positioning	Radiographic and anatomic study	Reproducibility of radiographic landmarks; no direct collision endpoint	Radiographic landmarks improved reproducibility of femoral tunnel placement.	Use landmarks to standardize the entry point, then verify the relationship to the ACL tunnel.
**TECHNICAL TUNNEL-AVOIDANCE OR CONTROL STRATEGIES**					
Mitrousias 2023 [19]	Anatomic ACLR + modified Lemaire LET	Technical note	Arthroscopic visualization of the ACL femoral tunnel during LET drilling	Direct visualization was proposed to detect impending breach or intersection.	Use real-time arthroscopic confirmation when a separate LET tunnel is drilled near the ACL tunnel.

Study	Procedure or construct	Method or design	Tunnel-interaction assessment	Key finding or technical implication	Application to LET planning
Hopper 2022 [26]	Modified Lemaire LET	Technical report	Low-profile onlay knotless suture anchor; no LET tunnel	The technique was proposed as a tunnel-sparing option with limited hardware prominence.	Consider onlay anchor fixation when bone stock or prior tunnels make another femoral socket undesirable.
Bechis 2024 [30]	Modified Lemaire LET + ACLR	Technical report	Indirect suspensory fixation through the ACL femoral device	The construct avoided a separate LET femoral implant or tunnel.	Consider indirect fixation when compatible with the ACL device and graft/tensioning plan.
Manze 2025 [36]	ACLR + modified Lemaire LET	Technical report	Single femoral tunnel with over-the-loop technique	A single-tunnel construct was proposed to eliminate tunnel convergence and reduce implant use.	Consider a shared-tunnel strategy only when graft routing, fixation security, and tensioning can be controlled.
Wasdev 2023 [42]	LET + ACLR	Technical note	Anterior/proximal guide-pin direction with arthroscopic control	Landmark-guided pin placement plus arthroscopic confirmation was proposed to reduce convergence.	Verify both entry point and trajectory before tunnel completion; redirect the pin if the ACL tunnel is threatened.

* Evidence derived from ALL reconstruction or non-modified-Lemaire lateral augmentation and used only for geometric or biomechanical extrapolation. Direct evidence measured actual or modeled tunnel/hardware interaction; indirect evidence informed position, isometry, or tunnel-sparing strategy without a direct collision endpoint. Technical reports describe proposed operative strategies and do not establish comparative superiority. Abbreviations: ACL, anterior cruciate ligament; ACLR, anterior cruciate ligament reconstruction; ALL, anterolateral ligament; CT, computed tomography; LET, lateral extra-articular tenodesis.

Table S5. Proposed minimum reporting checklist for LET femoral fixation studies.

Reporting item	Minimum information to report	Recommendation
LET procedure and graft	State the named LET technique or modification, graft source, graft width/length, route relative to the LCL, and whether combined with ALL reconstruction.	Recommended
Fixation family	Classify fixation as inlay, onlay, indirect/suspensory, or an alternative construct.	Recommended
Exact implant or fixation device	Report device type, manufacturer, material, and size when available (for example, screw, staple, anchor, all-suture anchor, or suspensory button).	Recommended
Primary versus revision setting	Specify primary or revision ACLR, prior femoral tunnels/hardware, tunnel widening, bone loss, and staged revision status.	Recommended
ACL femoral-tunnel characteristics	Report ACL tunnel drilling technique, aperture location, diameter, length, orientation, and retained hardware relevant to LET planning.	Recommended when applicable
LET femoral entry point	Describe the relationship to the lateral epicondyle, LCL, Kaplan fibers, posterior/proximal landmarks, and/or radiographic coordinates.	Recommended
Tunnel or socket dimensions	Report diameter and depth for every LET/ALL femoral tunnel or socket.	Recommended when applicable
Drilling direction	Report axial and coronal trajectory, anterior/proximal/distal orientation, and guide angle or guide system.	Recommended when applicable
Knee flexion during drilling	State the exact knee-flexion angle or range during LET/ALL femoral tunnel or socket creation.	Recommended when applicable
Knee position during fixation	State knee-flexion angle and varus/valgus and tibial-rotation conditions during fixation.	Recommended
Graft tensioning method	Report manual or device-assisted tension, measured load when used, incremental button tensioning, and/or anchor pretensioning.	Recommended
Collision-control strategy	Describe preoperative CT/simulation, fluoroscopy, guide-pin checks, arthroscopic or direct tunnel visualization, and any intraoperative redirection.	Recommended when applicable
Outcome timing and follow-up	Report assessment time points and minimum/mean follow-up for clinical, imaging, or biomechanical outcomes.	Recommended
Complications and reoperations	Report hardware irritation/removal, tunnel violation or convergence, overconstraint, infection, graft failure, and revision surgery.	Recommended
Clinical, imaging, or biomechanical endpoints	Define failure, pivot shift, laxity, PROMs, CT/radiographic criteria, load to failure, stiffness, failure mode, and overconstraint measures as applicable.	Recommended

This author-proposed checklist was derived from the reporting gaps identified in the present review and has not been formally validated. Abbreviations: ACLR, anterior cruciate ligament reconstruction; ALL, anterolateral ligament; CT, computed tomography; LCL, lateral collateral ligament; LET, lateral extra-articular tenodesis; PROMs, patient-reported outcome measures.
